# Effect of V/Mo Atomic Ratio on the Microstructure and Mechanical Properties of MoVCuN Coatings

**DOI:** 10.3390/ma17010229

**Published:** 2023-12-31

**Authors:** Haijuan Mei, Cihong Lin, Yuhang Li, Youqu Shen, Qiuguo Li, Rui Wang, Wenjun Zeng, Wenbao Mei, Weiping Gong

**Affiliations:** 1 Guangdong Provincial Key Laboratory of Electronic Functional Materials and Devices, Huizhou University, Huizhou 516007, China; 18927306664@163.com (C.L.); liyuhang02171030@163.com (Y.L.); yqshen@hzu.edu.cn (Y.S.); liqiuguo10@163.com (Q.L.); 2 School of Mechanical Engineering, Guilin University of Aerospace Technology, Guilin 541004, China; wanrui@guat.edu.cn; 3 Hunan Yongshan Lithium Industry Co., Ltd., Ningxiang 410203, China; zengwenjun@brunp.com.cn; 4 Shenzhen Qiling Image Technology Co., Ltd., Shenzhen 518000, China; meiwb@kimagex.com

**Keywords:** microstructure, mechanical properties, ion source assisted

## Abstract

To improve the gas ionization ratio, the Mo-V-Cu-N coatings were deposited by pulsed dc magnetron sputtering with assistance from an anode layer ion source, and the influence of the V/Mo atomic ratio was explored with regard to the microstructure and mechanical properties of the coatings. The findings of this study indicated that the MoVCuN coatings exhibited a solid solution phase of FCC B1-MoVN with a prominent (220) preferred orientation, and the deposition rate was found to decrease from 4.7 to 1.8 nm/min when the V/Mo atomic ratio increased. The average surface roughness of the MoVCuN coatings gradually decreased, and the lowest surface roughness of 6.9 nm was achieved at a V/Mo atomic ratio of 0.31. Due to the enhanced ion bombardment effect, the coatings changed from a coarse columnar to a dense columnar crystal structure, and promoted grain refinement at higher V/Mo atomic ratios, contributing to a gradual improvement in the compressive residual stress, hardness and adhesion strength of the coatings.

## 1. Introduction

As a promising self-lubricating coating, Mo-Cu-N coatings have received extensive research in recent years [1,2,3,4]. Because of the formation of lubricating oxides of MoO_3_ and CuMoO_4_ (MoO_3_ + CuO→CuMoO_4_), Mo-Cu-N coatings demonstrated exceptional tribological properties, including a low coefficient of friction and high resistance to wear, particularly at room temperature [5,6,7]. However, the wear rate of the Mo-N coatings was comparatively high at 500 °C as a result of severe oxidation at high temperatures; this restricted the application of self-lubricating coatings at high temperatures [8,9]. A multi-component structure was formed by incorporating V into Mo-N-based coatings in order to further enhance the tribological properties. Due to the solution strengthening, this addition not only increased the hardness, but also enhanced the wear resistance and high-temperature oxidation resistance. Wang et al. [10] reported that the MoN-V (22 at.%) coating demonstrated the highest hardness and the best wear resistance, as well as the lowest friction coefficient at 700 °C, which was mainly associated with the formation of lubricious glaze layers of V_2_O_5_ and MoO_3_. Similarly, the addition of V into the Mo-Cu-N coatings also improved the coating hardness and wear resistance [11,12]. Due to the synergistic lubrication effect of MoO_3_, CuMoO_4_, and V_2_O_5_, an excellent wear performance was achieved at room temperature. With increasing temperature, the wear resistance of the coatings was effectively enhanced with higher V contents.

Utilizing magnetron sputtering (MS), a typical physical vapor deposition (PVD) technique, transition metal nitrides with smooth surface were effectively deposited. However, the deposition rate is constrained by the low gas ionization ratio [13,14,15]. In order to improve the gas ionization ratio, an auxiliary ion source, including an anode layer ion source (ALIS) [16,17,18], end-Hall ion source [19,20], and Kaufman ion source [21,22], was proposed. The ion source utilized the orthogonal electromagnetic fields to increase the collision frequency between the electrons and gas atoms or molecules in the vacuum chamber, thereby increasing the gas ionization rate. With an increase in the power of the ion source, both the sp^3^ carbon content and deposition rate were increased for the BN [23] and α-CN_X_ [24] coatings deposited by ALIS-assisted radio-frequency magnetron sputtering. By varying the ion source discharge current, the hardness and adhesion were also enhanced for the TiN [25] coatings prepared by ALIS-assisted magnetron sputtering. ALIS-assisted high-power impulse magnetron sputtering (HIPIMS) was also employed to deposit the TiN_X_ coatings at various substrate bias voltages in an effort to enhance the gas ionization ratio [26]. Thus, with the assistance of ALIS, the comprehensive properties of the hard coatings can be further improved, mainly focusing on the deposition of binary coatings, such as BN, CN_X_, and TiN coatings.

In previous studies, the MoVCuN coatings were deposited by HIPIMS, which primarily emphasized the effect of deposition parameters, including the nitrogen partial pressure [27], and the charge voltage [28]. However, few studies report on the multi-component coatings deposited by pulsed dc magnetron sputtering (PDCMS) with ALIS assistance. In this study, to improve the gas ionization ratio, the MoVCuN coatings were deposited by ALIS-assisted PDCMS, and the influence of the V/Mo atomic ratio on the microstructure evolution, surface roughness, residual stress, and mechanical properties of the coatings were systematically investigated.

## 2. Experimental Details

### 2.1. Coating Deposition

**Figure 1:**
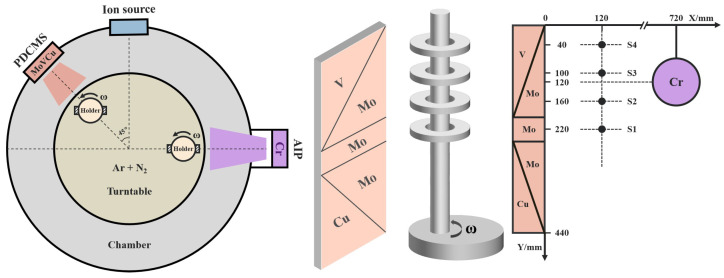
Schematic figure of the deposition system and sample position. The vertical positions of samples S1, S2, S3, and S4 correspond to 220 mm, 160 mm, 100 mm, and 40 mm, respectively. The center position of the Cr target corresponds to 120 mm.

The MoVCuN coatings were prepared on polished WC-Co cemented carbides and 316 L stainless steels by ion-source-assisted pulsed dc magnetron sputtering. the coatings deposited on the cemented carbide substrates were used for microstructure characterization and mechanical properties testing, while the coatings deposited on the stainless steel substrates were used for residual stress testing. The schematic figure of the Mo-V-Cu (99.9% purity, 69 mm × 443 mm) and Cr (99.99% purity, Ø100 × 20 mm) target positions is shown in Figure 1, and the vertical distance of the sample varied from 40 to 220 mm. All the substrates were dried prior to their installation on the substrate carrier following a 20 min ultrasonic cleaning procedure utilizing acetone and alcohol. Then, the chamber was pumped up to 4.0 × 10^−3^ Pa prior to deposition, with substrate temperature maintained at 200 °C. The substrates were subjected to Ar^+^ bombardment for 15 min at a 40% duty cycle and −1000 V bias voltage in order to eliminate the surface contaminants. To improve the adhesion strength between the coating and substrate, arc ion plating (AIP) with a Cr target was used to deposit a thin CrN sublayer for 5 min, operating at a target current of 100 A and a substrate bias voltage of −150 V. Then, this was followed by the deposition of MoVCuN coatings using pulsed dc magnetron sputtering with ALIS assistance for 500 min, operating at 1.5 kW target power and a duty cycle of 75%, and an ion source power of 0.6 kW. A mixed gas of N_2_ (10 sccm) and Ar (35 sccm) was introduced, the total gas pressure was controlled at 0.5 Pa by adjusting throttle valve, and the substrate holder was rotated at 3 rpm during deposition.

### 2.2. Coating Characterization

Scanning electron microscopy (SEM, Nano430, Amsterdam, The Netherlands) with EDS was utilized to characterize the morphologies and chemical composition of the coatings. The coating thickness was directly measured through the cross-section images observed by SEM; then, the deposition rate could be calculated. The characterization of crystal structure was performed using X-ray diffraction (XRD, Bruker D8 advance, Karlsruhe, Germany) with Cu *K_α_* radiation and a scanning angle ranging from 30° to 90° at a step size of 0.02°. The preferable orientation was determined using the texture coefficient formula [29]:

(1) $$T_{\left(hkl\right)}\;=\;\frac{I_{\left(hkl\right)}/I_{0(hkl)}}{\frac{1}{n}\sum_{n=1}^{n}I_{\left(hkl\right)}/I_{0(hkl)}}$$

where *I_0(hkl)_* and *I_(hkl)_* refer to the relative standard intensity of the MoN powder and intensity of the measured *(hkl)* peak, respectively, and n refers to the reflection number. The determination of surface roughness was carried out by atomic force microscope (AFM, Bruker dimension Icon, Karlsruhe, Germany), operating in a contact mode with a 5 × 5 μm^2^ scan area. The characterization of the chemical structure was performed using X-ray photoelectron spectroscopy (XPS, Escalab 250Xi, Waltham, MA, USA) with Al *K_α_* X-ray source. The residual stress was measured using a film stress tester (FST-1000, Supro Instruments, Shenzhen, China), in accordance with the substrate curvature method based on Stoney’s equation [30]:

(2) $$\sigma_{s}=\frac{E_{s}}{6\left(1-\upsilon_{s}\right)}\frac{h_{s}^{2}}{h_{c}}\left(\frac{1}{R}-\frac{1}{R_{0}}\right)$$

where *E_s_* and *υ_s_* refer to the Elastic modulus and Poisson’s ratio of the substrate, respectively. *h_s_* and *h_c_* denote the thickness of the substrate and coating, respectively. *R*_0_ and *R* represent the curvature radius of the substrate before and after deposition, respectively. The hardness and elastic modulus were assessed using a nanoindentation tester (NHT^2^, CSM, Peseux, Switzerland), and a minimum of five indentations were made per sample. The adhesion strength was assessed by a scratch tester (RST, CSM, Peseux, Switzerland) operating at a maximal load of 100 N and a scratch length of 3 mm. Optical microscopy (OM) was then employed to examine the scratch images, and at least three scratches were made for each sample.

## 3. Results and Discussion

### 3.1. Microstructure

**Figure 2:**
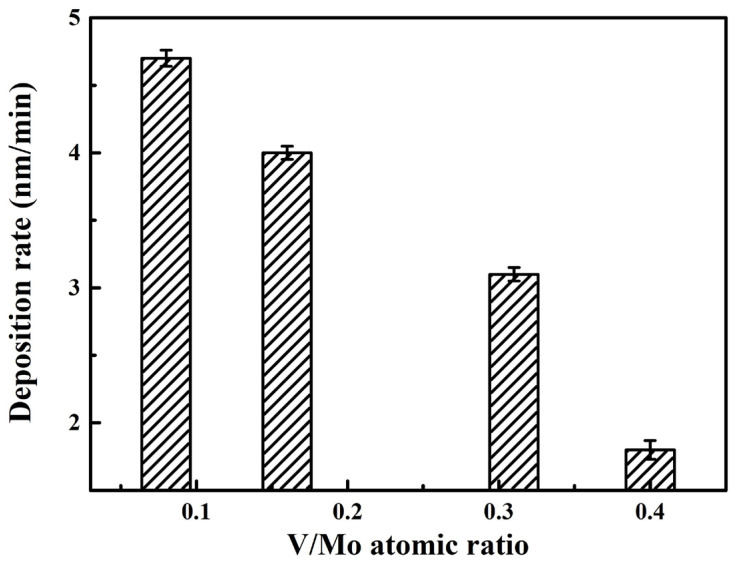
Deposition rate of MoVCuN coatings at various V/Mo atomic ratios.

Table 1 presents a list of the chemical composition of MoVCuN coatings. Due to the special design of the relative position between the target and sample (in Figure 1), coatings with varying compositions can be obtained. With the decrease in the sample distance from 220 to 40 mm, the Mo content gradually decreased from 54.8 to 39.5 at.%, and the Cu content slightly decreased from 0.6 at.% to 0.3 at.%. Correspondingly, the contents of V and N gradually increased from 4.4 at.% and 40.2 at.% to 15.9 at.% and 44.3 at.%, respectively. The calculated N/(Mo + V) atomic ratio gradually increased from 0.68 to 0.80, implying that all the coatings were substoichiometric, and the V/Mo atomic ratio gradually increased from 0.08 to 0.40. With an increase in the V/Mo atomic ratio, the coating thickness gradually decreased, along with a gradual decrease in the deposition rate from 4.7 to 1.8 nm/min, as shown in Figure 2. Such a decrease in the deposition rate can be related to the different sputtering yields of metal targets. Among the Mo-V-Cu targets, the Cu and Mo metal targets with higher sputtering yields were preferentially sputtered during the deposition process, and the V metal target had the lowest sputtering yield [31].

**Figure 3:**
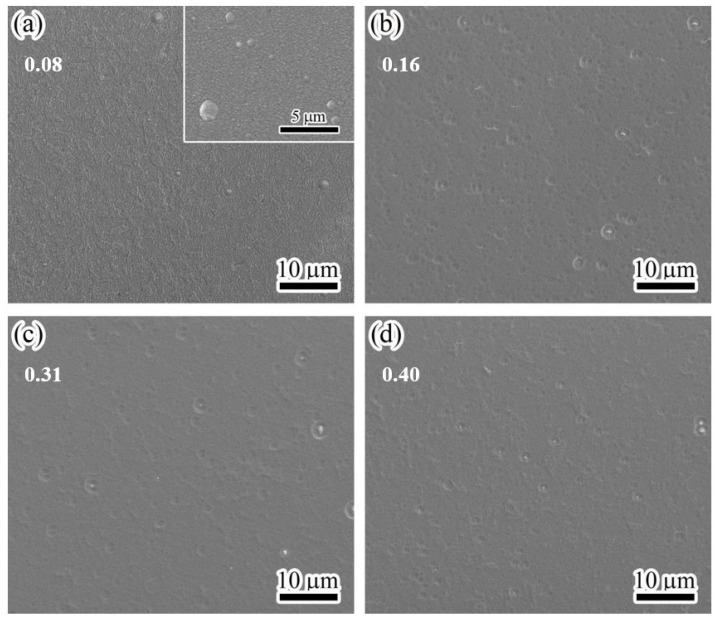
Surface micrographs of MoVCuN coatings at various V/Mo atomic ratios: (**a**) 0.08, (**b**) 0.16, (**c**) 0.31, (**d**) 0.40. Inset is the high-magnification image.

**Figure 4:**
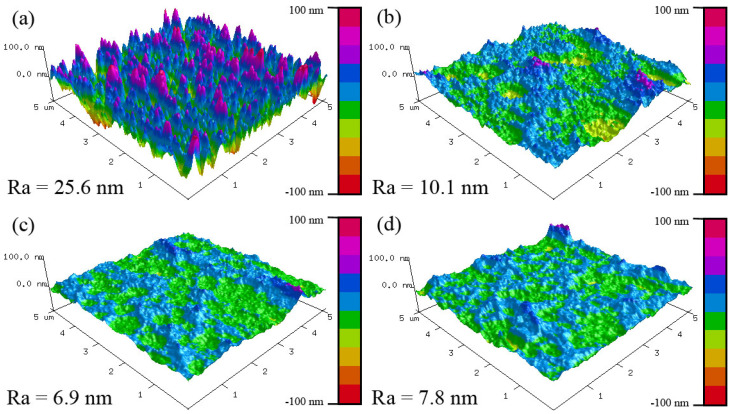
Surface roughness of MoVCuN coatings at various V/Mo atomic ratios: (**a**) 0.08, (**b**) 0.16, (**c**) 0.31, (**d**) 0.40.

Figure 3 shows the surface morphologies of the MoVCuN coatings at various V/Mo atomic ratios. In Figure 3a, the coating has a rough surface and is covered with many small bulging peaks. In addition, some large microparticles can be observed in the inset high-magnification image. Generally, the advantage of magnetron sputtering is that the coating surface is very smooth, without any microparticles [10,27]. These microparticles were mainly introduced through the deposition of CrN interlayer using arc ion plating (AIP) [7]. During the deposition process, the arc spots generated by arc discharge evaporated the local area of Cr target with a high temperature, and some large metal droplets flew towards the substrate surface before forming some large microparticles, showing a typical growth defect of AIP. However, when the V/Mo atomic ratio increased above 0.16, the coating surfaces became much smoother and denser. Furthermore, due to the combined effects of etching and ion bombardment, obvious shallow pits and small microparticles developed on the coating surfaces. During ion-source-assisted deposition, the ionization rate of sputtered atoms was increased, and the ions accelerated to bombard the coating surface under the substrate bias voltage. When the V/Mo atomic ratio increased, a greater number of accelerated V ions were directed towards the coating surface under substrate bias voltage, thereby enhancing the ion bombardment effect. Prior to coating deposition, the polished cemented carbide substrate showed a low surface roughness of 2.7 nm [12]. However, the surface roughness of the coatings increased sharply. In Figure 4a, a much rougher surface can be observed, which is consistent with the findings obtained by SEM. As the V/Mo atomic ratio increased from 0.08 to 0.31, the surface roughness gradually decreased from 25.6 ± 1.3 nm to 6.9 ± 0.4 nm, and then reached 7.8 ± 0.7 nm at a high V/Mo atomic ratio of 0.40. The variation in the surface roughness was mainly related to the enhanced ion bombardment at higher V/Mo atomic ratios. With the increase in the V/Mo atomic ratio, the thickness of MoVCuN coatings gradually decreased, which also led to a decrease in the surface roughness. In addition, due to the variation in sample position, the thickness of the CrN sublayer first increased and then decreased. As discussed above, the microparticles were mainly introduced by the CrN sublayer; thus, the increase in CrN layer thickness would increase the surface roughness.

**Figure 5:**
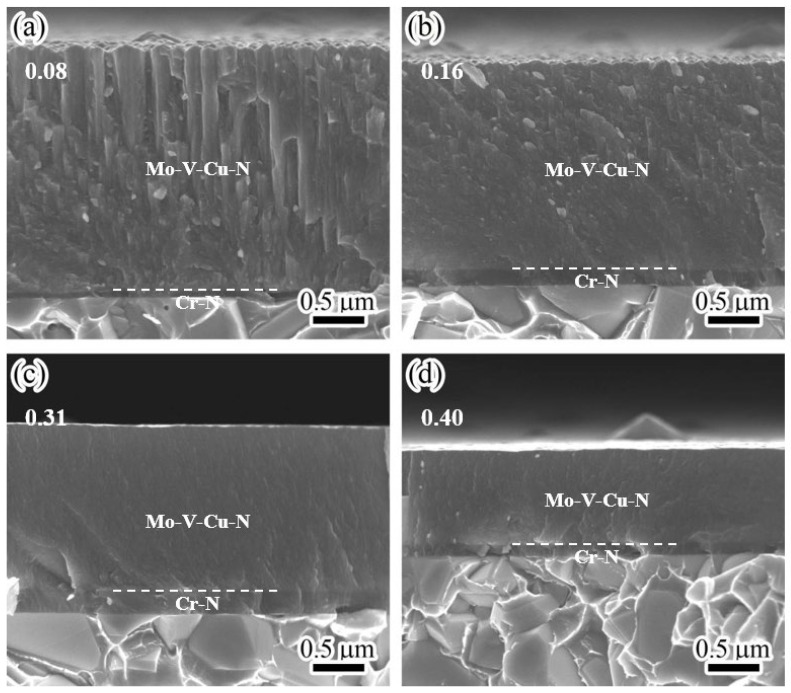
Cross-sectional micrographs of MoVCuN coatings at various V/Mo atomic ratios: (**a**) 0.08, (**b**) 0.16, (**c**) 0.31, (**d**) 0.40.

Figure 5 displays the cross-sectional morphologies of MoVCuN coatings at various V/Mo atomic ratios. It can be seen that two layers formed in the cross-sections, including the thin interlayer of CrN and the top layer of MoVCuN. Because of the special design of the two target positions (in Figure 1), the thickness of the CrN layer initially increased from 0.07 to 0.22 μm, and then decreased to 0.12 μm, whereas the thickness of the MoVCuN layer gradually decreased from 2.34 to 0.88 μm. At the interlayer interface, all the coatings exhibited good adhesion without any cracks. In Figure 5a, the MoVCuN coating showed a coarse columnar crystal structure. The growth in these coarse columnar crystals ran through the entire thickness of the coating and formed a typical V-shaped columnar crystals structure, contributing to the rough surface morphology depicted in Figure 3a. A similar columnar structure was also demonstrated for the Mo-V-N coatings [10]. With an increase in the V/Mo atomic ratio, the columnar structure became much denser, and the continuous growth of the columnar crystal was gradually interrupted. Compared to the Mo target, the V target has a lower sputtering yield and deposition rate. However, for the magnetron sputtering, due to the limited ionization ratio (10~20%), the sputtered V atoms have a higher metal ionization ratio per unit deposition time. Thus, the increased V/Mo atomic ratios resulted in higher metal ionization ratios of Mo and V atoms. These ions were accelerated to bombard the coating surface under substrate bias voltage, thereby intensifying the ion bombardment effect. Strong ion bombardment increased the mobility of surface adatom during deposition, resulting in the formation of a dense and fine columnar crystal morphology [12,32].

**Figure 6:**
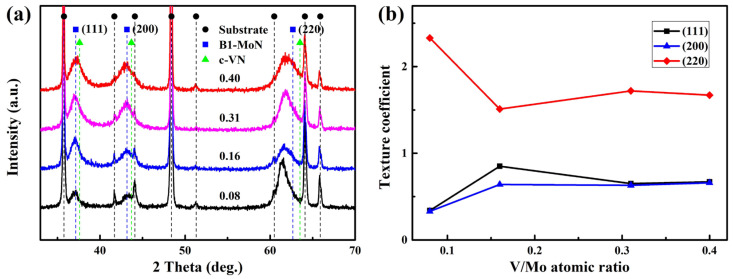
XRD pattern (**a**) and texture coefficient (**b**) of MoVCuN coatings.

Figure 6 shows the XRD pattern and texture coefficient of the MoVCuN coatings at various V/Mo atomic ratios. As shown in Figure 6a, three broad diffraction peaks in the coatings appeared at about 37.1°, 43.2°, and 61.5°, corresponding to the (111), (200), and (220) planes of the face-center-cubic (FCC) phase, respectively. Due to the similar diffraction peak positions, the diffraction peaks of the thin layer of Cr-N would be covered by that of the top layer of Mo-V-Cu-N. In addition, some sharp diffraction peaks were observed, corresponding to the diffraction peaks in the WC-Co cemented carbide substrate. The major phase of Mo-N was observed in the XRD patterns as a result of the relatively high Mo content (39.5~54.8 at.%) in the coatings. As a result of their comparable atomic radius, V atoms showed a tendency to partially substitute for Mo atoms in the Mo-N lattice, thereby facilitating the formation of a solid solution phase of Mo-V-N [7]. A similar solid solution phase of FCC Mo-V-N was also found in the Mo-V-N [10] and V_1-x_Mo_x_N coatings [33]. However, the absence of a diffraction peak associated with the Cu phase in XRD patterns necessitates additional XPS analysis, as the Cu atoms were present in relatively low contents (0.3~0.6 at.%). Related research has indicated that, in the Mo-Cu-N [2] and Mo-V-Cu-N [28] coatings, Cu atoms predominantly existed in an amorphous state when the Cu content was below 11 at.%. In Table 2, the lattice parameters of the coatings were calculated from the (111), (200), and (220) planes in the XRD patterns by using Gaussian fitting [34]. With an increase in the V/Mo atomic ratio, the lattice parameter varied in a small range of from 4.215 to 4.204 Å, corresponding to the FCC B1-MoN phase, a metastable phase characterized by a lattice parameter ranging from 4.20 to 4.27 Å [35]. Thus, the MoVCuN coatings showed a single solid solution phase consisting of FCC B1-MoVN. In previous study [12], due to the low N contents (23.9~42.5 at.%), the γ-Mo_2_(V)N phase with lattice parameters of 4.162~4.197 Å formed in the MoVCuN coatings. Furthermore, the grain sizes of the (111), (200), and (220) peaks in the B1-MoVN phase were estimated by Scherrer formula [36], as listed in Table 2. As the V/Mo atomic ratio increased, the average grain size gradually decreased from 7.0 to 5.1 nm, indicating that grain refinement occurred, which could be related to the enhanced ion bombardment effect [37]. In addition, as the thickness increased, the crystallinity of the coating increased. Thus, a decrease in the thickness of MoVCuN coatings also contributed to a decrease in the grain size. In Figure 6b, based on the (111), (200), and (220) peaks, the evaluation of the preferred orientation was conducted by the texture coefficient *T_(hkl)_* using Equation (1). The (220) plane demonstrated the highest texture coefficient, ranging from 1.51 to 2.33. This indicated that all the coatings exhibited a prominent (220) preferred orientation. According to the growth kinetic model [38], the (220) plane with the most open channeling directions has a higher probability of survival than the (111) and (200) planes due to the anisotropy of the collision effect. A similar (220) preferred orientation was also observed for the AlTiVCuN coatings deposited by ALIS-assisted magnetron sputtering [39] and HIPIMS [40].

**Figure 7:**
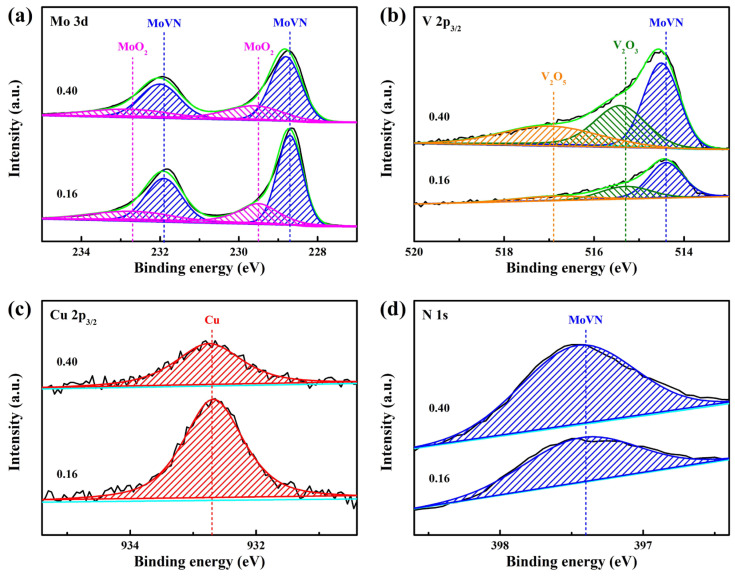
Fitted XPS spectra of MoVCuN coatings: (**a**) Mo 3d, (**b**) V 2p_3/2_, (**c**) Cu 2p_3/2_, (**d**) N 1s.

Figure 7 illustrates the fitted XPS spectra of the MoVCuN coatings at various V/Mo atomic ratios. The deconvolution of the asymmetric Mo 3d peak into four peaks, as shown in Figure 7a, can correspond to the major peaks in MoVN (Mo 3d_5/2_: 228.7 eV, Mo 3d_3/2_: 231.9 eV), along with the minor peaks in MoO_2_ (Mo 3d_5/2_: 229.5 eV, Mo 3d_3/2_: 232.7 eV) [41]. The minor oxide peaks can be caused by the slight oxidation of the coating surface. In Figure 7b, the fitted spectra of V2p_3/2_ comprised three sub-peaks: a major peak of MoVN at 514.4 eV, and two oxide peaks of V_2_O_3_ and V_2_O_5_ at 515.3 eV and 516.9 eV, respectively. In Figure 7c, only one peak was identified, at about 932.7 eV, in the spectra of Cu 2p_3/2_, which could belong to the metallic Cu [2]. This provided evidence that the Cu atoms in the coatings existed as metallic species rather than existing in nitride form. Similarly, the N 1s spectra only showed a single peak, possibly the MoVN peak, at 397.4 eV binding energy. Consistent with the XRD data mentioned earlier, this demonstrated the formation of a Mo-V-N solid solution phase. With an increase in the V/Mo atomic ratio, the peak areas of both Mo 3d and Cu 2p_3/2_ spectra decreased, while the peak areas of V 2p_3/2_ and N 1s spectra significantly increased, which was consistent with the variation in the chemical composition of the coatings, as listed in Table 3. Due to the surface adsorption of pollutants and trace oxides, a large amount of O and C elements were also detected.

### 3.2. Mechanical Properties

**Figure 8:**
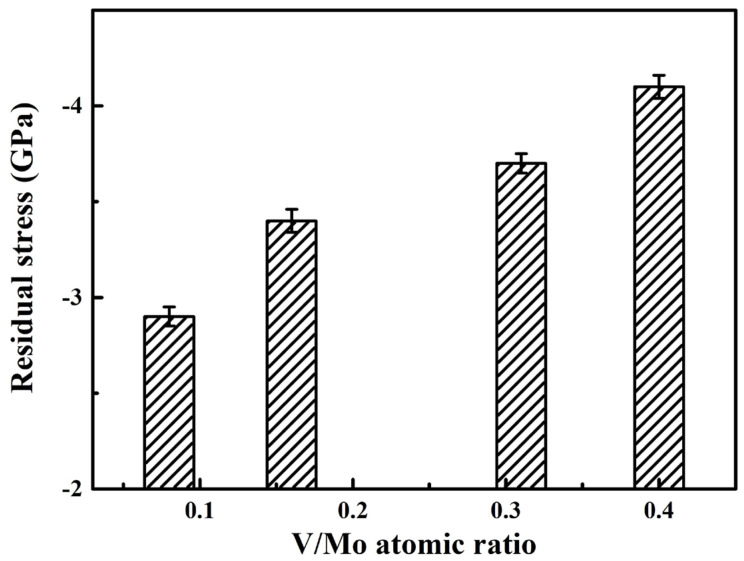
Residual stress of MoVCuN coatings at various V/Mo atomic ratios.

Generally, the residual stress of the coatings or films includes thermal stress and intrinsic stress. Thermal stress is usually caused by the discrepancy in thermal expansion between the substrate and coating. As for the nitride coatings, due to their lower coefficient of thermal expansion, compressive thermal stress occurs. As for the sputter-deposited coatings, the intrinsic stress is usually caused by the deposition conditions [42], such as working temperature, gas pressure, and substrate bias voltage. Figure 8 presents the residual stress of the coatings at various V/Mo atomic ratios. The negative value of the residual stress calculated for the coatings suggests the formation of compressive residual stress in the coatings. With an increase in the V/Mo atomic ratio, the compressive residual stress gradually increases from 2.9 GPa to 4.1 GPa, which can be explained by several different factors. First, at higher V/Mo atomic ratios, the ion bombardment effect is enhanced, increasing the defect concentration of the coatings, leading to an increase in compressive residual stress [43]. Second, the increase in compressive residual stress may be associated with a slight decrease in the content of Cu, as a result of the ductile Cu phase presented in the coatings. Similar findings were found for the Mo-Cu-N [3] and Al-Ti-V-Cu-N [40] coatings: the relaxation of compressive residual stress occurs at higher Cu contents. Finally, the increase in residual stress may also be associated with the decrease in the coating thickness in Figure 5. For thinner coatings (≤4 μm), the residual stress was found to decrease with the increase in coating thickness [44].

**Figure 9:**
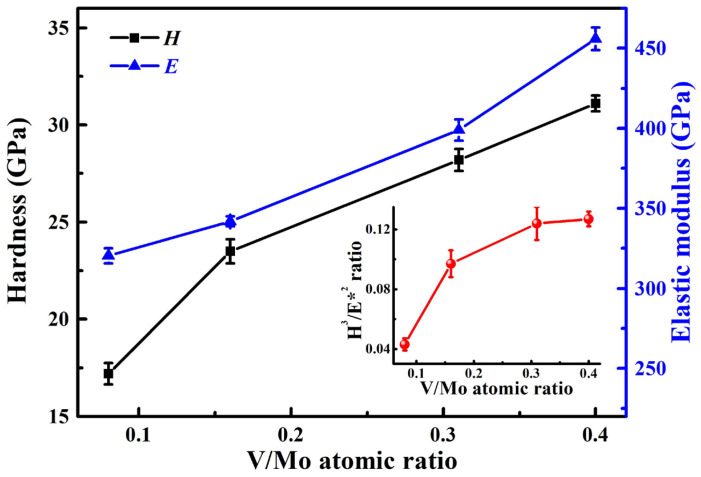
Hardness, elastic modulus, and *H*^3^/E*^2^ ratio of MoVCuN coatings.

Figure 9 displays the elastic modulus and hardness of the coatings at various V/Mo atomic ratios. To reduce the effect of the cemented carbide substrate and CrN sublayer, the maximum indentation depth was maintained at less than 10~15% of the thickness of MoVCuN coatings. At a low V/Mo atomic ratio of 0.08, the MoVCuN coating demonstrated a low hardness of 17.2 GPa and a low elastic modulus of 320.3 GPa, which can be attributed to the formation of a coarse columnar crystal structure within the coating. With an increase in the V/Mo atomic ratio, the hardness and elastic modulus gradually increased from to 31.1 and 455.9 GPa, respectively. This could be explained by many factors, including the microstructure evolution, and variations in Cu content, residual stress and grain size. First, the increase in hardness can be ascribed to the microstructure densification. With increasing V/Mo atomic ratio, the coatings transformed from a coarse into a dense columnar structure due to the enhanced ion bombardment effect. Furthermore, the enhanced hardness was also associated with the solution-strengthening effect of the B1-MoVN phase. Similar results have been reported for the Mo-V-N [10] and Mo-V-Cu-N [12] coatings with increases in the V content. Second, as a soft metal, the slight decrease in Cu content (0.6~0.3 at.%) was also correlated with enhanced hardness. It was also discovered that the hardness of TiAlN/Cu coatings progressively decreased when the Cu content increased, ranging from 0 to 1.4 at.% [45]. Third, the increase in compressive residual stress also contributed to an improvement in hardness. By restraining the grain sliding and grain rotation, the high compressive residual stress could help to resist plastic deformation in the coatings and increase the coating hardness. Finally, the increased hardness was also related to a decrease in the grain size. Based on the Hall–Petch effect, the hardness of BN coatings was effectively improved by decreasing the grain size [46,47]. With an increase in the V/Mo atomic ratio, the *H*^3^/E*^2^ ratio gradually increased from 0.04 to 0.13, implying that the ability to resist crack initiation and the propagation of the coatings was improved.

**Figure 10:**
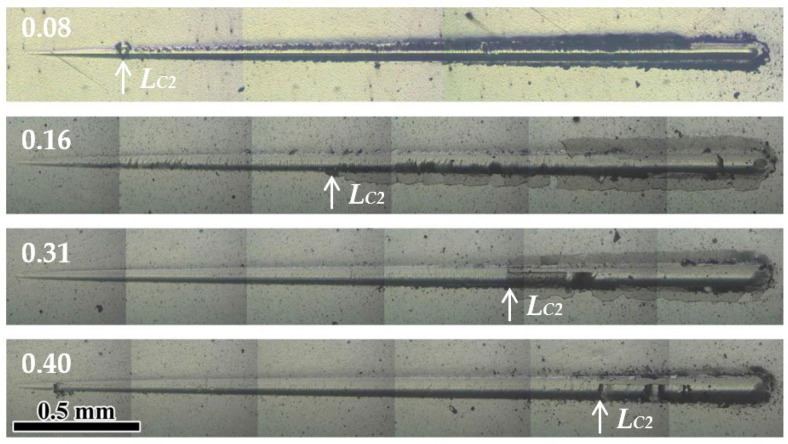
Scratch images of MoVCuN coatings at various V/Mo atomic ratios.

**Figure 11:**
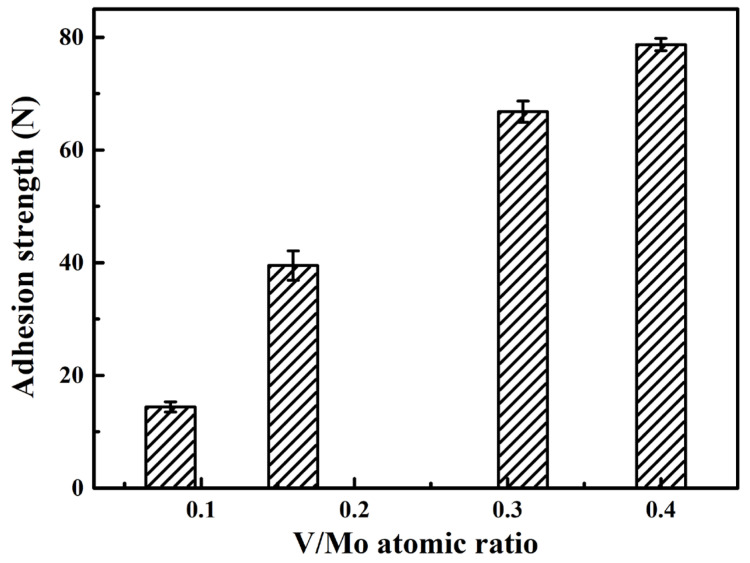
Adhesion strength of MoVCuN coatings at various V/Mo atomic ratios.

Figure 10 presents scratch images of the coatings at various V/Mo atomic ratios. Generally, the adhesive failure mode *L_C_*_2_, characterized by adhesive chipping at the track edges, was employed to assess the adhesion strength between the substrate and coating. At a low V/Mo atomic ratio of 0.08, the adhesive chipping occurred at the initial stage of the scratch test, and numerous adhesive fragments were dispersed along the track edge, indicating a low adhesion strength, which could be caused by the low *H*^3^/E*^2^ ratio of 0.04. Similar results were also found for the MoVCuN [12] and AlTiVCuN [48] coatings with low *H*^3^/E*^2^ ratios. However, when the V/Mo atomic ratio increased to above 0.16, smooth scratch morphologies can be observed in the initial stages, and adhesive chipping started in the middle or later stages, implying a significant improvement in the adhesion strength of the coatings. In Figure 11, with increasing the V/Mo atomic ratio, a gradual increase in the adhesion strength was observed from 14.4 to 78.7 N, which was mainly attributed to an increase in the *H*^3^/E*^2^ ratio. The increase in adhesion strength was also associated with the presence of Cu content in the coatings. The addition of Cu adversely influenced the coating/substrate bond, resulting in a lower adhesion strength and lower energy for the Cu-containing coatings [3]. Moreover, the increase in adhesion strength was also related to an increase in the thickness of the CrN sublayer used to improve the adhesion strength between the coating and substrate.

## 4. Conclusions

In this study, the MoVCuN coatings were deposited using pulsed dc magnetron sputtering with ALIS assistance, and the relationship between the microstructure evolution, mechanical properties, surface roughness, and the residual stress was investigated. The primary findings can be summarized as follows:

(1) As the V/Mo atomic ratio increased, the deposition rate sharply decreased from 4.7 to 1.8 nm/min, the average surface roughness of the coatings gradually decreased.
(2) The MoVCuN coatings exhibited a solid solution phase of FCC B1-MoVN with a strong (220) preferred orientation. As the V/Mo atomic ratio increased, the coatings transformed from a coarse to a dense columnar crystal structure, and promoted grain refinement.
(3) With an increase in the V/Mo atomic ratio, the ion bombardment effect was enhanced, contributing to a gradual increase in the compressive residual stress, hardness, and adhesion strength of the coatings.

## Figures and Tables

**Table 1:** Chemical composition of MoVCuN coatings according to EDS analysis; error ± 0.2 at.%.

Sample	Chemical Composition (at.%)				N/(Mo + V) Ratio	V/Mo Ratio	Thickness (μm)
	Mo	V	Cu	N			
S1	54.8	4.4	0.6	40.2	0.68	0.08	2.3
S2	50.5	8.0	0.4	41.1	0.70	0.16	2.0
S3	44.1	13.7	0.4	41.8	0.72	0.31	1.6
S4	39.5	15.9	0.3	44.3	0.80	0.40	0.9

**Table 2:** Lattice parameters and grain sizes of Mo-V-Cu-N coatings.

Plane	Lattice Parameter a_0_ (Å)				Grain Size (nm)			
	0.08	0.16	0.31	0.40	0.08	0.16	0.31	0.40
(111)	4.199	4.197	4.197	4.186	8.2	6.7	6.7	6.1
(200)	4.185	4.194	4.192	4.199	5.4	5.1	5.1	5.1
(220)	4.261	4.243	4.238	4.227	7.4	4.8	5.4	4.2
Mean	4.215	4.211	4.209	4.204	7.0	5.5	5.7	5.1
Stdev	0.040	0.027	0.025	0.021	1.4	1.0	0.9	1.0

**Table 3:** Chemical composition of MoVCuN coatings according to XPS analysis.

Sample	V/Mo Ratio	Chemical Composition (at.%)					
		Mo	V	Cu	N	O	C
S2	0.16	37.4	3.6	1.2	15.5	30.2	12.1
S4	0.40	28.2	5.7	0.5	22.6	30.5	12.5

## Data Availability

The data presented in this study are available on request from the corresponding author.
